# Treatment of Central Airway Stenosis With Self‐Expanding Y Stents: Easy and Innovative Technique With a Single Wire Guide

**DOI:** 10.1111/1759-7714.70197

**Published:** 2026-03-06

**Authors:** Gaetana Messina, Giuseppe Vicario, Davide Gerardo Pica, Massimo Ciaravola, Francesca Capasso, Vincenzo Di Filippo, Beatrice Leonardi, Riccardo Vinciguerra, Rosa Mirra, Maria Antonietta Puca, Noemi Maria Giorgiano, Anna D'Agostino, Martina Robustelli, Giovanni Vicidomini, Alfonso Fiorelli

**Affiliations:** ^1^ Thoracic Surgery Unit Università Degli Studi della Campania “Luigi Vanvitelli”, Napoli Campania Italy; ^2^ Anesthesiology Unit Università Degli Studi della Campania “Luigi Vanvitelli”, Napoli Campania Italy

**Keywords:** airway obstruction, bronchial stenosis, bronchoscopy, endoluminal disease, external compression, lung cancer, mediastinal tumors, self‐expanding metallic Y‐stents (SEMS)

## Abstract

**Objectives:**

Central airway obstruction (CAO) involves the narrowing of the trachea, carina, and main bronchi. This study describes a technique for placing a self‐expanding metallic Y‐stent using a single guidewire for the palliative management of inoperable malignant stenosis near the carina, evaluating its efficacy and safety.

**Materials and Methods:**

We conducted a retrospective analysis of all patients with severe malignant carinal stenosis who were treated with a customized self‐expanding metallic Y‐stent at our institution between January 2020 and December 2024. In all cases, the left bronchial branch of the stent was positioned using the Seldinger technique with a single guidewire.

**Results:**

The single‐guidewire Seldinger technique simplified the procedure, resulting in a significantly shorter stent placement time (38 vs. 51 min; *p* < 0.0001) and reduced general anesthesia time (53 vs. 71 min; *p* ≤ 0.0001) compared to a double‐guidewire approach. Furthermore, it minimized the number of required X‐ray exposures (0–1 vs. 4–5 images; *p* < 0.0001) and lowered the risk of guidewire dislodgement. No immediate complications were reported.

**Conclusion:**

The placement of a self‐expanding Y‐stent using a single left‐sided guidewire is an efficacious and feasible approach for maintaining airway patency in patients with severe malignant carinal stenosis, offering a simpler and more efficient procedural alternative.

## Graphical abstract

A single guidewire Seldinger technique enables accurate placement of a customized self expanding Y stent in malignant carinal stenosis. The wire stabilizes the left main bronchus, where carinal angles are more variable, allowing sequential deployment of the bronchial limbs followed by the tracheal limb. Compared with a doubleguidewire method, this approach significantly reduces procedure and anesthesia times, limits fluoroscopic exposures, and maintains a high technical success rate without immediate complications.

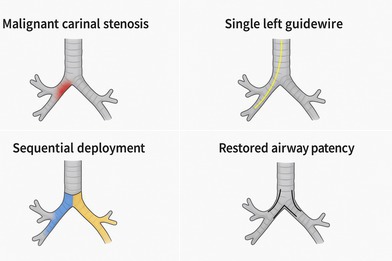

## 1 Introduction

Central airway obstruction (CAO) involves the narrowing of the trachea, carina, and main bronchi. The most common cause of central airway stenosis is lung cancer, but other malignancies, such as distant metastases from a primary tumor, esophageal cancer and lymphoma, may also be responsible. Severe carinal stenosis, defined as a narrowing of the tracheal lumen of approximately 75% (residual tracheal lumen < 5 mm in diameter), precisely at the bifurcation between the trachea and the main bronchi, a location that corresponds to the carina, certainly represents a very serious scenario, being a life‐threatening clinical condition. Surgical resection is usually contraindicated for several reasons. This study describes a technique for placing a self‐expanding metallic Y‐stent using a single guidewire in the management of patients with malignant airway obstruction near the carina, as a palliative, effective, and safe method in patients with inoperable airway stenosis [1, 2, 3].

## 2 Materials and Methods

**FIGURE 1:**
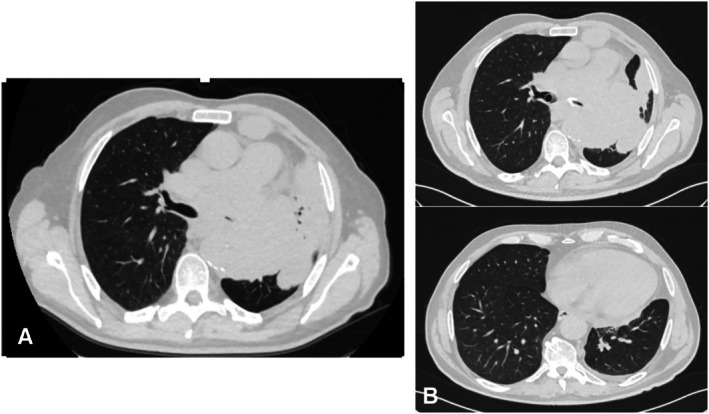
Multislice spiral computed tomography (MSCT) scan of the chest were performed to provide reliable data for stent selection and size customization. (A) Pre stent‐placement and (B) post stent placement.

This retrospective study included all patients with malignant carinal stenosis treated with a customized Y‐shaped metal self‐expanding stent at the University of Campania Luigi Vanvitelli in Naples between January 2020 and December 2024. Patients underwent bronchoscopy and high‐resolution chest computed tomography (HRCT) beforehand, to provide reliable data for customizing the stent size (Figure 1). Preoperative evaluations, conducted by the anesthesiologist and the thoracic surgeon, included chest CT, total body PET‐CT, and an endoscopic examination which confirmed neoplastic infiltration at the carinal and endobronchial levels. The dimensions of the self‐expanding Y‐stent were customized for each patient based on a comprehensive pre‐procedural assessment of the airways. This involved precise measurements obtained from HRCT scans, which provided detailed anatomical data on the length of the stenotic segment, the diameters of the trachea and main bronchi proximal and distal to the obstruction, and the carinal angle. These CT‐based measurements were subsequently integrated with and validated by direct visual assessment and measurement during flexible bronchoscopy. The bronchoscopic evaluation was crucial for confirming the dynamic characteristics of the stenosis and ensuring accurate in vivo sizing [4, 5, 6].

**FIGURE 2:**
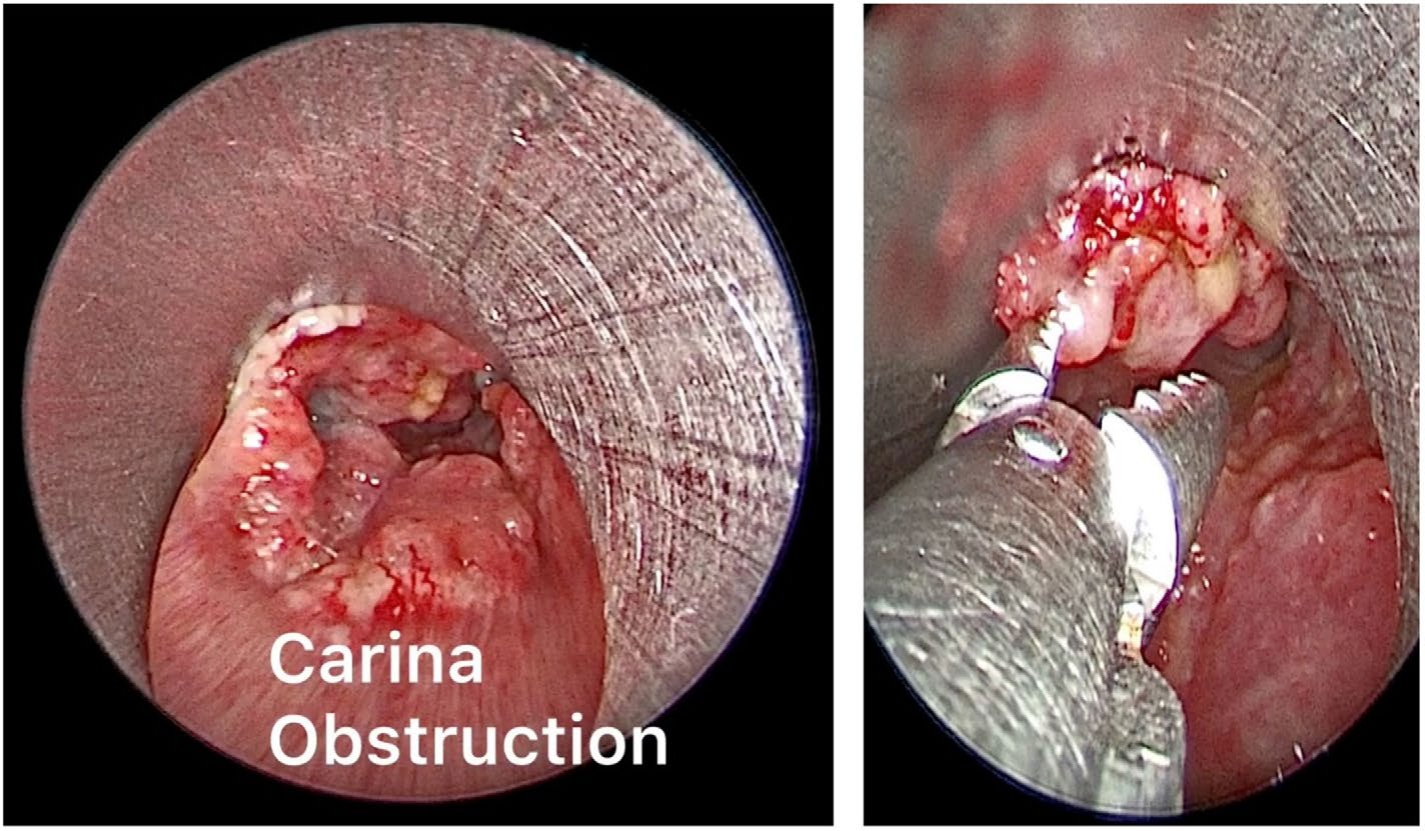
Mechanical debulking, electrosurgery, and laser ablation were performed before stent placement.

**FIGURE 3:**
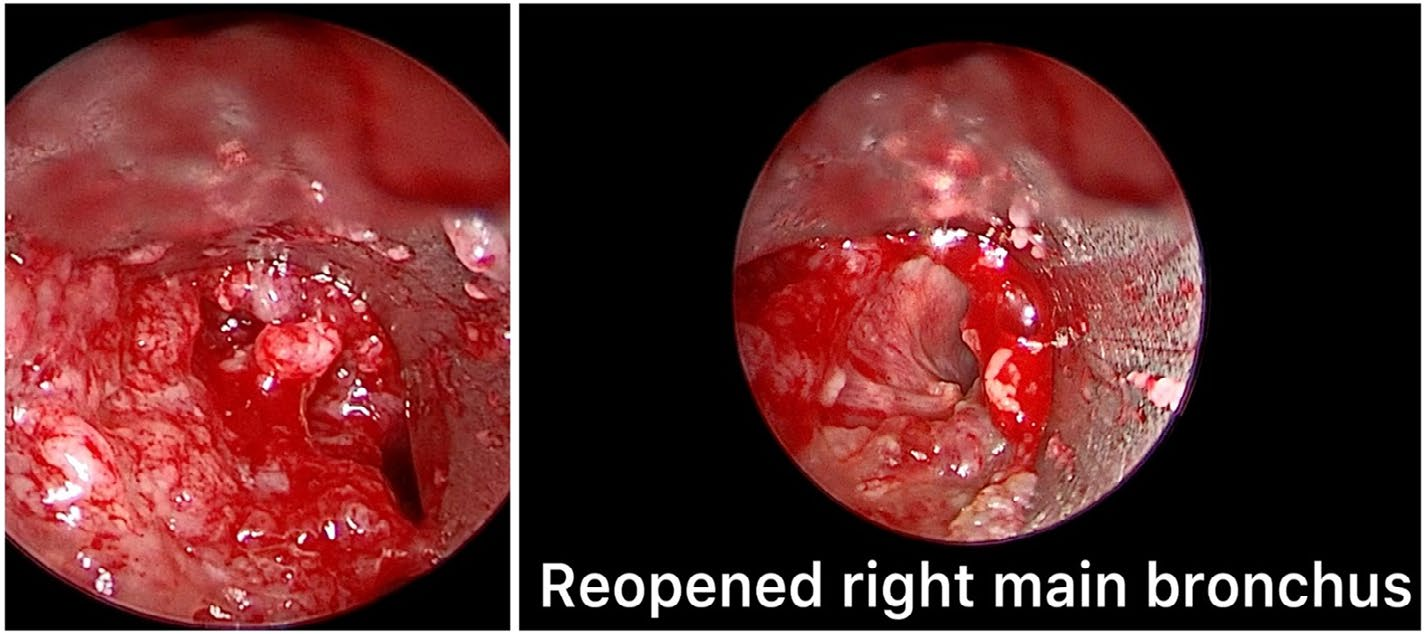
A satisfactory recanalization was obtained for the right bronchus, while on the left the neoplasm dislocated the axis of the main bronchus and prevented a complete clearing.

**FIGURE 4:**
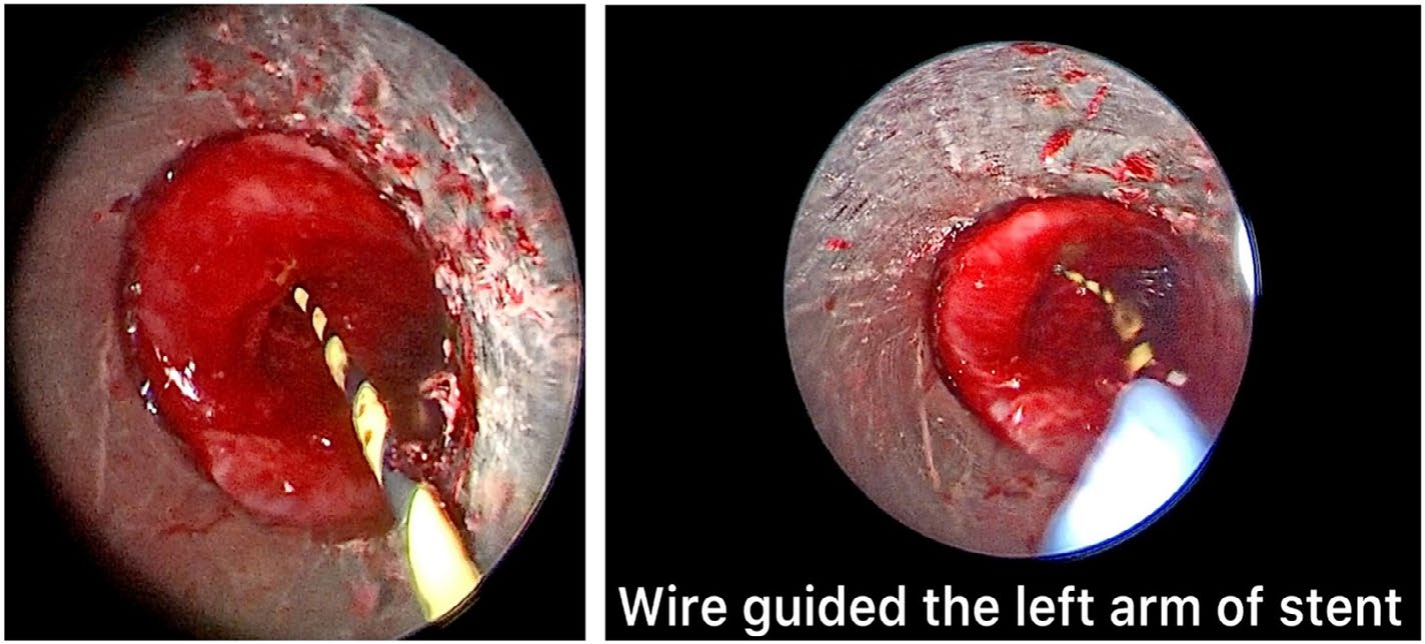
A 0.035‐in. left wire guidewire was placed into the left mainstem bronchus through the working channel of the flexible bronchoscope past the stenotic lesion.

**FIGURE 5:**
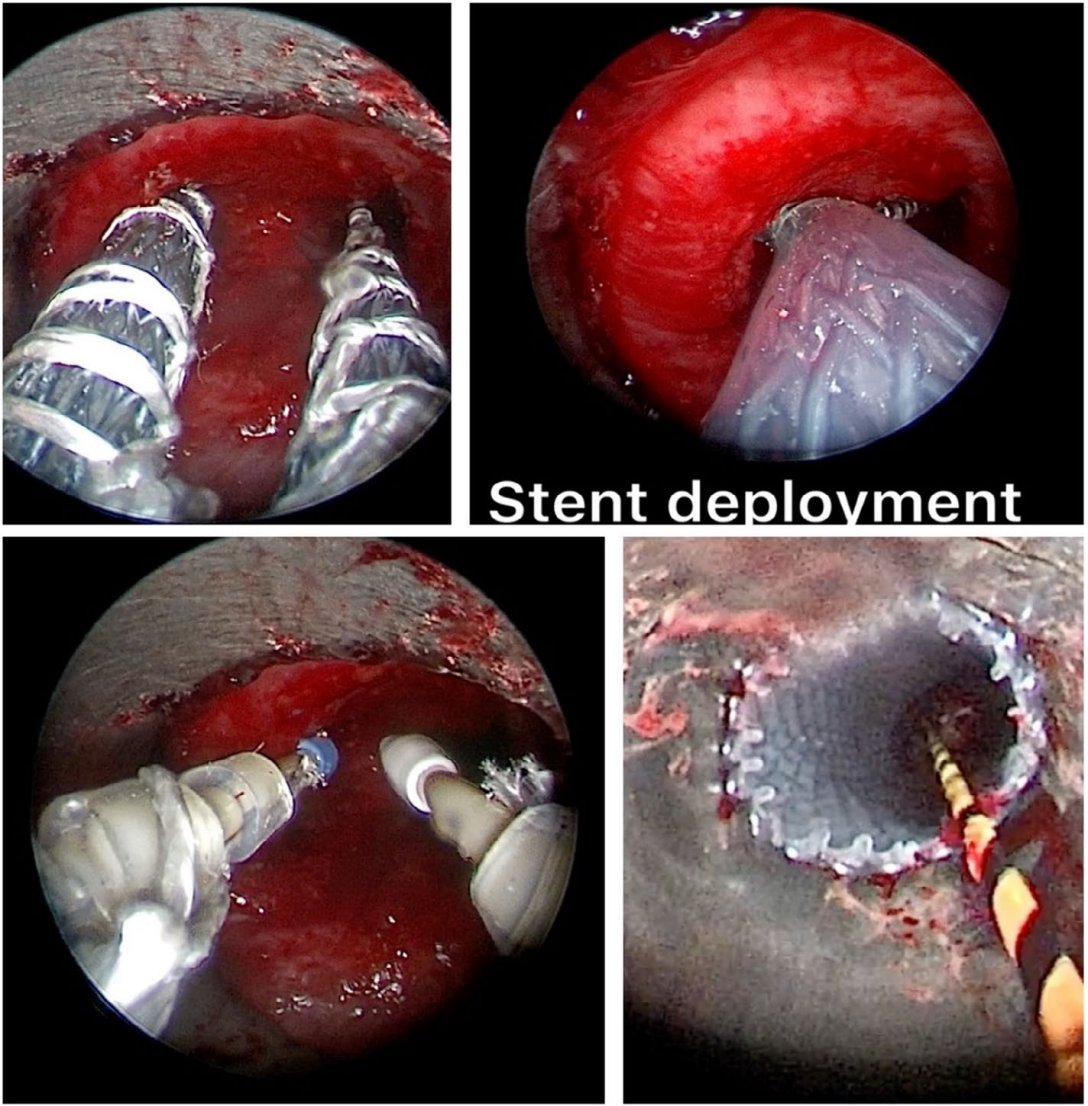
The placement of the prosthesis is done by slowly rotating it to the left and right until both bronchial branches, are advanced, with the left branch advancing along metal guides to the correct position.

**FIGURE 6:**
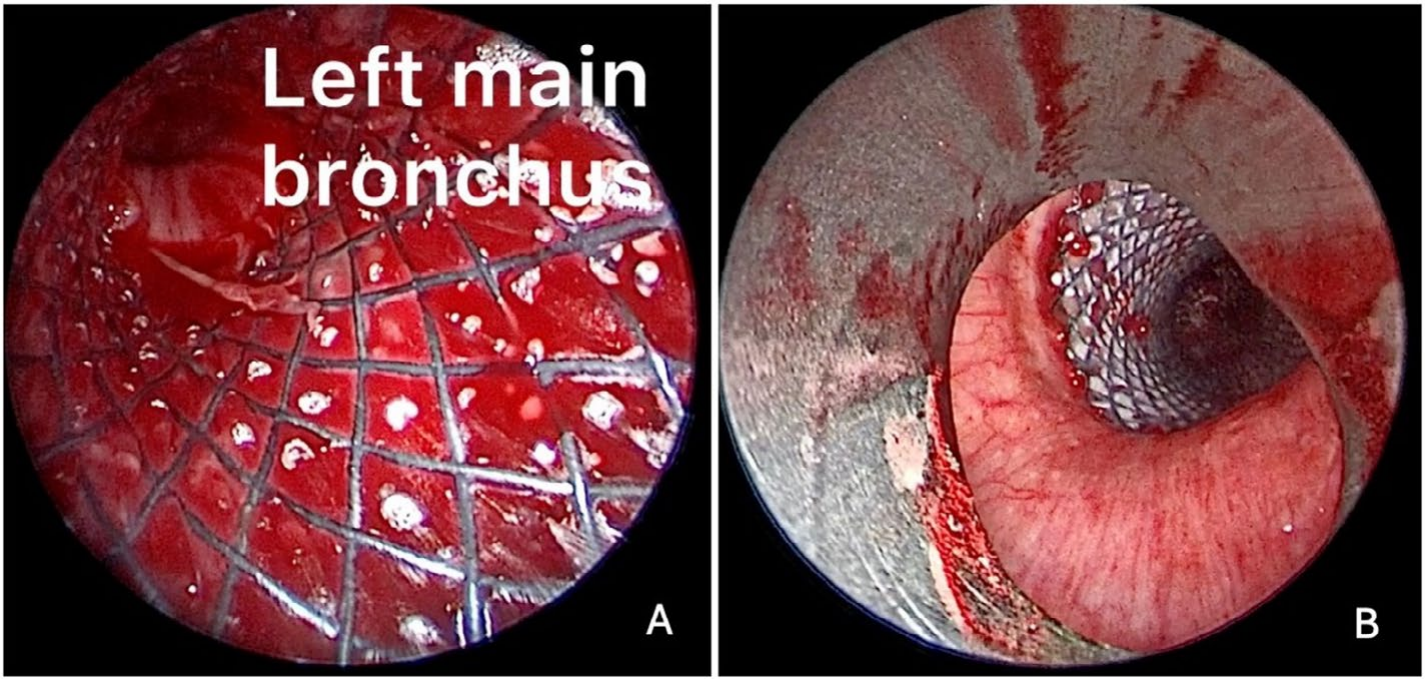
(A) Left main bronchus post stent placement using the Seldinger technique. (B) Trachea after Y‐stent placement.

The placement of the Y‐shaped stents was performed in rigid bronchoscopy under general anesthesia with high‐frequency jet ventilation, thus ensuring better tolerability and safety in patients. Before stent placement, other interventional procedures were performed such as mechanical debulking and laser ablation (Figure 2). Mechanical debulking and laser ablation are performed as a preparatory maneuver for Y‐prosthesis placement in cancer patients with carinal stenosis, defined as a narrowing of the tracheal lumen of approximately 75% (residual tracheal lumen < 5 mm in diameter). These recanalization maneuvers are generally more effective in the right main bronchus due to its anatomical configuration; specifically, the right main bronchus forms a 20° angle with the trachea, positioning it in near‐direct continuity (Figure 3). Conversely, the left main bronchus forms an angle of approximately 40°–50° relative to the trachea. This greater vertical inclination, which is often further accentuated by neoplastic tissue infiltration, complicates adequate recanalization. This anatomical challenge also dictates the procedural strategy: the guide wire is introduced into the left main bronchus to guide the initial positioning of the branch of the Y‐prosthesis that is more challenging to place [7, 8, 9]. The Y‐tracheal stent was specifically designed to overcome stenosis in the carinal region. This device is constructed from a nitinol mesh and covered by a polymeric membrane. This covering is deliberately omitted for the distal 5 mm of the right main bronchus, a feature designed to prevent its closure in case of inadvertent oversizing of the stent. This self‐expanding Y‐shaped metal stent is available in different sizes and configurations, including 16 and 20 mm diameters, as well as an 18 mm configuration with 12 and 14 mm bronchi. However, it is not adjustable in length. The stents possess several key features: an atraumatic tip, an anatomical conical shape, high radial force and flexibility, and X‐ray visibility via tantalum markers. The stents are mounted in their own 8 mm diameter delivery system, featuring a single‐wire mechanism [10, 11, 12]. Before introduction through the rigid bronchoscope, the bronchial branches of the Y‐prosthesis were exposed after initial retraction of the introducer sheath, then a 0.035‐in. guidewire was placed in the left mainstem bronchus through the working channel of the flexible bronchoscope beyond the stenotic lesion, after which the flexible bronchoscope was retracted, leaving the guidewire in place in the left mainstem bronchus (Figure 4). The bronchial branches of the Y‐shaped stent were exposed after initial retraction of the introducer sheath, prior to introduction through the rigid bronchoscope. The guidewire was then introduced into the left branch of the Y‐prosthesis and the stent loaded in its introducer sheath (9 mm external diameter) was inserted into the rigid bronchoscope and advanced to the carina under direct vision [13, 14, 15], this procedure was performed in real time under direct vision. Once the correct position of the guidewire was verified in correspondence with the left bronchus, SEMS placement was started. The stent placement was performed by gently and slowly rotating it to the left and right until both bronchial branches were positioned, advancing the left branch along the metal guidewire and the right branch into the right main bronchus until the correct position (Figure 5); finally ensuring the opening of the bronchial part first, followed by the tracheal part, until all parts of the stent were completely opened. The bronchial branches are color‐coded: blue for the left and yellow for the right. After being positioned, the stent was further pushed in correspondence with the carina, so that the bronchial branches of the stent were placed in their respective positions (Figure 6). After stent placement, the delivery system and guidewire were removed quickly. Correct positioning of the bronchial branches was confirmed by fluoroscopy or under direct visualization via an ultrathin flexible bronchoscope (2.8 mm) [16]. All patients were nebulized and treated with antibiotics for 3 days after treatment to prevent infection, relieve chest pain, and thin sputum. The effect of stent placement was evaluated by the improvement in Hugh–Jones (HJ) grade and SaO_2_ before and after treatment [17]. All patients underwent chest CT scans the day after stent placement, at 1 month, and every 2–3 months after treatment to evaluate long‐term patency of the stent. The health status of each patient was assessed by contacting patients and/or their family members monthly after treatment [18]. We also compared the 33 patients treated with Y prostheses with a single guide wire with 28 patients (16 men and 12 women; median age 63 years; age range 55–65) in which we used two guidewires treated from 2018 to December 2023. The most common cause of stenosis was nonsmall cell lung cancer (10 patients; 36%); small cell lung cancer (three patients; 11%); and esophageal cancer (three patients; 6%); lymphoma (two patients; 9%); and distant metastases from a primary tumor (one patient; 3%). All patients presented with progressive dyspnea (28 patients; 100%), stridor (26 patients; 92%), hemoptysis (18 patients; 64%), cough (26 patients; 93%), and chest pain (eight patients; 29%). Six patients (30%) had minor complications, such as cough (two patients; 33%), formation of granulation tissue at the stent site (four patients; 12%), increased secretions (five patients; 15%), or dyspnea (four patients; 12%). Thirteen patients (65%) had stent restenosis due to tumor growth and 10 of them (77%) required further interventions; two patients (15%) had stent fractures, and one patient (8%) had a tracheoesophageal fistula due to progression of esophageal cancer. One patient died 1 day after stent placement for respiratory failure caused by acute pulmonary embolism. Eight subjects (29%) developed granulation tissue at the stent margins. In two of these subjects (25%), granulation tissue ablation was performed with electrocautery. No stent‐associated migration was observed. All patients were advised to nebulize acetylcysteine (0.3 g, 3 mL) and saline at least twice daily after Y‐stent placement to reduce excessive secretions (Tables 1 and 2).

**TABLE 1:** Patients in which we used a single guidewire.

Variables	*N* = 33
Age range (year)	30–83
Mean duration of Y‐stent placement (min)	38 min (31 ± 45)
Gender	Male 20 (60%) Female 13 (40%)
Histology, *n* (%)	NSCLC 25 (76%) SCLC 2 (6%) Esophageal cancer 2 (6%) Lymphoma 3 (9%) Metastasis 1 (3%)
Symptoms, *n* (%)	Dyspnea 33 (100%) Stridor 27 (82%) Hemoptysis 21 (64%) Cough 31 (94%) Chest pain 9 (27%)
Degree of luminal obstruction, *n* (%)	2 (50%–74%) → 18 (55%) 3 (75%–89%) → 10 (30%) 4 (90%–100%) → 5 (15%)
Respiratory support (*n*)	Room air breathing 0 Low O_2_ therapy via cannula 0 High O_2_ therapy via mask 31 Invasive ventilation 2

**TABLE 2:** Patients in which we used two guidewires.

Variables	*N* = 28
Age range (year)	55–65 years
Gender	Female 16 (57%) Male 12 (43%)
Mean duration of Y‐stent placement (min)	51 min (45 ± 56)
Mean duration of general anesthesia	71 min (65 ± 78)
Number of X‐rays	4/5
Symptoms, *n* (%)	Dyspnea 4 (12%) Hemoptysis 5 (15%) Cough 2 (33%) Formation granulation tissue 12 (43%)

## 3 Statistical Analysis

A significant difference was found comparing the group of patients in which we used a single guidewire (33) with the group of patients in which we used two guidewires (28), a *p* < 0.05 was considered statistically significant. We have found Y‐stent placement with single guidewires is certainly simpler than Y‐stent placement with two guidewires, since less time is needed (38 min vs. 51 min; *p* < 0.0001), less time of general anesthesia (53 min vs. 71 min; *p* ≤ 0.0001), and a fewer number of X‐rays (1 or 0 X‐rays in the first group; 4–5 X‐rays in the second group; *p* < 0.0001).

## 4 Results

Between January 2020 and December 2024, a total of 33 patients (20 men and 13 women; median age, 61 years; age range, 30–83 years) with severe malignant carinal stenosis were enrolled and treated with a self‐expanding Y‐stent at the Thoracic Surgery Unit of the University of Campania Luigi Vanvitelli in Naples. The most common cause of malignant carinal stenosis was nonsmall cell lung cancer (25 patients; 76%); esophageal cancer (two patients; 6%); small cell lung cancer (two patients; 6%), distant metastases from a primary tumor (one patient; 3%); and lymphoma (three patients; 9%). All patients presented with progressive dyspnea (33 patients; 100%), hemoptysis (21 patients; 64%), stridor (27 patients; 82%), chest pain (nine patients; 27%), and cough (31 patients; 94%). Stent placement was technically successful in 32 patients, resulting in a technical success rate of 97% (32/33). The degree of luminal obstruction was classified as: grade 1, < 50% in no patients; grade 2, 50%–74% in 18 patients (55%); grade 2, 75%–89% in 10 patients (30%); and grade 4, 90%–100% in five patients (15%). Respiratory failure improved immediately after treatment, and all patients improved by at least one Hugh–Jones (HJ) grade after the procedure. Respiratory support was classified into four grades: room air breathing in no patients; low‐concentration oxygen therapy via cannula (FiO_2_ ≤ 33%) in no patients; high‐concentration oxygen therapy via mask (FiO_2_ ≥ 35%) in 31 patients; and invasive ventilation in two patients. Blood gas analysis before endoscopic treatment showed the following values: pH: 7.28 ± 2, pCO_2_: 53 ± 5 mmHg, pO_2_: 78.7 ± 5 mmHg, HCO_3_act: 26.4 ± 3 mmol/L, BE: 3.2 mmol/L, sO_2_: 89.4% ± 5%, in HFNC FiO_2_ 60% con P/F: 100; while after the placement of the prosthesis, the patient's respiratory efficiency improved significantly: pH: 7.36 ± 3, pCO_2_: 40.5 ± 3 mmHg, pO_2_: 85.7 ± 4 mmHg, HCO_3_act: 26.4 ± 2 mmol/L, BE: 0.4 mmol/L, sO_2_: 94.4% ± 3 in HFNC FiO_2_: 45% con P/F: 154. The two patients (6%) who underwent invasive ventilation, were extubated immediately after the procedure. The mean duration of Y‐stent placement was 38 min (31 ± 45). Stent size was decided based on airway measurements by computed tomography and flexible bronchoscopy. Respiratory support after the Y‐stent placement was used as an indirect parameter of symptom improvement: room air breathing in 15 patients (49%), low‐concentration oxygen therapy via cannula (FiO_2_ ≤ 33%) in 13 patients (33%), high‐concentration oxygen therapy via mask (FiO_2_ ≥ 35%) in four patients (13%), and invasive ventilation in one patient (3%). Y‐stent placement was unsuccessful in one patient because the caliber of the stent was smaller than the tracheal lumen, so the undersized stent was removed using an Alligator Grasping Forcep, grasping the prosthesis near the distal metal part of the prosthesis, then rotating the clip counterclockwise, to bend the prosthesis on itself, reducing the tracheal lumen of the prosthesis, thus allowing its removal [19, 20, 21]. The duration of follow‐up ranged from 7, 15, 30, and 130 days, with a median of 57 days. Short‐term follow‐up (1 week after operation) data were acquired from three patients (9%), medium‐term follow‐up (1–3 months after operation) data were acquired from 26 patients (79%), and long‐term follow‐up (more than 3 months after operation) data were acquired from four patients (12%). Follow‐up data were based on bronchoscopic recordings and CT imaging. Short‐term complications (1 week after operation) were recorded in two patients (6%), medium‐term complications in 26 patients (79%) (1–3 months after operation), and long‐term complications in three patients (9%) (more than 3 months after operation) such as secretion retention and restenosis. Data on complications in the remaining two (6%) patients are not reported because they did not undergo further follow‐up. Comparing two groups of patients in which we used a single guidewire (33) with the group of patients in which we used two guidewires (28), we found shorter time needed for Y‐stent placement, less time of general anesthesia and a smaller number of X‐rays in the first group compared to the second group makes this method statistically significant (*p* < 0.0001).

## 5 Discussion

CAO is a very serious and potentially lethal condition, manifested by dyspnea, hemoptysis, and apnea in case of complete airway obstruction; however, compensatory mechanisms generally ensure adequate breathing, but if the central airway lumen is less than 5 mm, severe respiratory failure inevitably occurs. In case of acute respiratory compromise, emergency endoscopic treatment is required and the insertion of an endoluminal stent ensures immediate improvement of symptoms [22, 23, 24]. In case of carina involvement, a Y‐stent can be used. In 1972, Neville and colleagues (6) designed the first Y‐stent, a silicone stent. Over the years, airway stents have become increasingly sophisticated and today several models of Y‐stent are available, differing in material, stiffness, and insertion technique [25, 26, 27]. The self‐expanding metal Y‐stent, compared to the silicone stent, is easier to position and adapt to the tracheal and bronchial lumen, even in poor general conditions of the patient. Metal stents, thanks to their thin walls, have a larger internal diameter than silicone stents, facilitating the removal of secretions. A stent that is oversized compared to the stenosis can cause excessive radial force on the airway mucosa, causing excessive growth of granulation tissue and, less frequently, erosion or perforation, while an undersized stent can migrate. Self‐expanding metal stents are available in different sizes and can even be customized. The Y‐stent is made of nickel–titanium alloy; thanks to its memory effect, it returns to its original size and shape after positioning. In our study, we placed a self‐expanding and customized metal Y‐stent in all patients [28, 29, 30]. Self‐expanding Y‐stent provides more precise adaptation to the irregularities of airway stenosis, achieving satisfactory therapeutic effects and avoiding complications. The main stent‐related complication was stent restenosis caused by tumor growth, in the medium and long term in this study [31, 32, 33]. Correct measurements of length and diameter, position of the affected airway segment, distal and proximal diameter of adjacent healthy areas, and angles between the right and left mainstem bronchus certainly provide accurate and detailed information for the design of customized Y‐stent. In addition, multislice CT provides further detailed anatomical information of the distal airway [34, 35, 36]. Generally, Y‐stent placement with two guidewires may be associated with technical difficulties; despite appropriate precautions, guidewire crossing occurred. Therefore, in our study we demonstrated that the placement of the Y‐stent does not necessarily require the placement of two guidewires; since the right and left bronchial branches of the stent, once pushed out of the introducer sheath, form an angle almost equal to that of the tracheal carina. Therefore, since the right main bronchus is in direct continuity with the carina, the guidewire of the right bronchial branch of the Y‐stent is not necessary, being easily positioned. Otherwise, the use of a single guidewire, and precisely the left one, is useful, since the left main bronchus has a greater inclination with respect to the trachea; in the presence of neoplasms that infiltrate the carina, the inclination of the angle between the bronchus itself and the carina is further modified [37, 38, 39]. The placement of a single guidewire on the left allows the left branch to be positioned correctly more easily, avoiding incorrect positioning in the mediastinum with consequent injury to the bronchial wall, while ensuring adequate recanalization of the bronchial lumen and limiting all possible related complications [40, 41, 42]. While our findings are promising, several limitations of this study warrant consideration. Firstly, the relatively small number of patients from a single institution, along with its retrospective nature, may introduce biases and limit the broad applicability of our results. The comparative analysis, though revealing significant advantages for the single‐guidewire technique, would be strengthened by a prospective, randomized design. Furthermore, data on long‐term outcomes and patient survival were often incomplete. This unfortunately restricts our insight into the very long‐term performance of the stents. Finally, a formal assessment of quality‐of‐life improvements, a crucial endpoint in palliative therapy, was beyond the scope of this technical analysis. Future studies addressing these points will be valuable to further validate this technique [43, 44, 45].

## 6 Conclusion

The present study described the placement of a customized self‐expanding Y‐stent with a single guidewire and demonstrated its efficacy and feasibility for maintaining tracheal and mainstay bronchial patency in malignant carinal stenosis. This approach could be used as an alternative method before starting adjuvant therapy and final palliative therapy for symptom relief. The self‐expanding nitinol Y‐stent with a single guidewire is easy to place, and in some cases, buys time until the start of tumor‐specific therapy. Although surgery remains the “gold standard” treatment in these patients to avoid complications associated with stent implantation, if surgery is not possible, stent implantation represents a valid alternative therapeutic approach.

## Author Contributions

All authors contributed to the design of the study. G.M. and G.V. contributed to the conception of the study. D.G.P., V.D.F., and F.C. contributed to the drafting of the article. N.M.G. and B.L. contributed to data collection and imaging analysis. M.C., D.G.P., R.V., R.M., M.A.P., N.M.G., A.D., and M.R. participated in data analysis and interpretation and led the revision of the article. G.V. and A.F. supervised the study. All authors reviewed and approved the final manuscript.

## Funding

This work was supported by the University of Campania “L. Vanvitelli.”

## Ethics Statement

The authors are accountable for all aspects of the work in ensuring that questions related to the accuracy or integrity of any part of the work are appropriately investigated and resolved. The study was conducted in compliance with the principles of the Declaration of Helsinki; and the protocol was approved by the Ethics Committee of the University of “Luigi Vanvitelli” of Naples (32655/2021).

## Consent

Written informed consent was obtained from all participants during preoperative communication.

## Conflicts of Interest

The authors declare no conflicts of interest.

## Data Availability

Authors can confirm that all relevant data are included in the article.
